# The gut microbiome as a target for prevention and treatment of hyperglycaemia in type 2 diabetes: from current human evidence to future possibilities

**DOI:** 10.1007/s00125-017-4278-3

**Published:** 2017-04-22

**Authors:** Louise Brunkwall, Marju Orho-Melander

**Affiliations:** Department of Clinical Sciences in Malmö, Lund University Diabetes Centre, Lund University, Jan Waldenströms gata 35, 205 02 Malmö, Sweden

**Keywords:** 16S sequencing, Faecal microbiome, Genetics, Glycaemic control, Gut microbiome, Metagenomics, Metformin, Personalised nutrition, Probiotics, Review, Type 2 diabetes

## Abstract

The totality of microbial genomes in the gut exceeds the size of the human genome, having around 500-fold more genes that importantly complement our coding potential. Microbial genes are essential for key metabolic processes, such as the breakdown of indigestible dietary fibres to short-chain fatty acids, biosynthesis of amino acids and vitamins, and production of neurotransmitters and hormones. During the last decade, evidence has accumulated to support a role for gut microbiota (analysed from faecal samples) in glycaemic control and type 2 diabetes. Mechanistic studies in mice support a causal role for gut microbiota in metabolic diseases, although human data favouring causality is insufficient. As it may be challenging to sort the human evidence from the large number of animal studies in the field, there is a need to provide a review of human studies. Thus, the aim of this review is to cover the current and future possibilities and challenges of using the gut microbiota, with its capacity to be modified, in the development of preventive and treatment strategies for hyperglycaemia and type 2 diabetes in humans. We discuss what is known about the composition and functionality of human gut microbiota in type 2 diabetes and summarise recent evidence of current treatment strategies that involve, or are based on, modification of gut microbiota (diet, probiotics, metformin and bariatric surgery). We go on to review some potential future gut-based glucose-lowering approaches involving microbiota, including the development of personalised nutrition and probiotic approaches, identification of therapeutic components of probiotics, targeted delivery of propionate in the proximal colon, targeted delivery of metformin in the lower gut, faecal microbiota transplantation, and the incorporation of genetically modified bacteria that express therapeutic factors into microbiota. Finally, future avenues and challenges for understanding the interplay between human nutrition, genetics and microbial genetics, and the need for integration of human multi-omic data (such as genetics, transcriptomics, epigenetics, proteomics and metabolomics) with microbiome data (such as strain-level variation, transcriptomics, proteomics and metabolomics) to make personalised treatments a successful future reality are discussed.

**Electronic supplementary material:**

The online version of this article (doi:10.1007/s00125-017-4278-3) contains peer-reviewed but unedited supplementary material including a slideset of the figures for download, which is available to authorised users.

## Introduction

Accumulating evidence indicates the importance of the gut microbiota in the development of a multitude of complex human diseases, including type 2 diabetes. The intimate coevolution of the microbiota with mammalian hosts has created a coherent symbiotic relationship that directs and contributes to a plethora of essential physiological functions, such as energy metabolism, metabolic signalling, formation of the immune system, and regulation of integrity and mobility of the gut barrier. A paradigm shift in the research of gut microbiota in health and disease came after the development of PCR-based techniques for the characterisation and quantification of bacterial composition via sequencing of bacterial genes. In contrast to cloning-, blotting- or cultivation-based analyses, sequencing techniques enable a much more comprehensive mapping of bacteria, alongside investigation of their abundance in samples of interest, which in human gut microbiota studies have almost exclusively been faeces specimens. Thus, throughout this review, the term ‘gut microbiota’ refers to faecal microbiota. The two main approaches for next-generation sequencing of the microbiome are 16S ribosomal RNA gene amplicon sequencing and shotgun metagenomics sequencing, both of which have their strengths and weaknesses (see summary text box: gut microbiome analysis) [1]. The sequencing methods used in the studies discussed in this review are listed in ESM Table 1. Whilst considering the findings discussed in this review, we would like the readers to note that, in addition to the type of sequencing methods used, the results of gut microbiome analyses are affected by the methods used for sampling and storage and DNA extraction, the approaches and pipelines used for taxonomical classification of bacterial sequences, and the bioinformatics and statistical approach applied for data integration and analysis [1, 2].
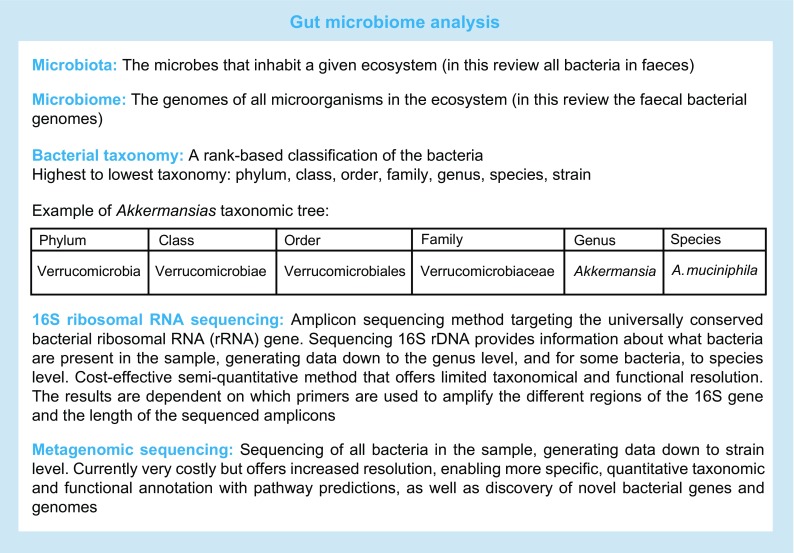


### What factors impact on the gut microbiota?

During 2016 the first two population-based gut microbiota studies were published. In an attempt to obtain a comprehensive picture of the factors that correlate with the human gut microbiota, Falony et al investigated the association between 503 clinical-, health- and lifestyle-related variables and the diversity and composition of gut microbiota among 1106 Belgian individuals and also in a replication cohort (Dutch LifeLines DEEP; *n* = 1135) [3]. They observed that stool consistency had the largest effect size on gut microbiota, while medication-, diet- and health-related variables majorly contributed to the variance in gut microbiota. Zhernakova et al performed a more detailed analysis of the Dutch LifeLines DEEP cohort using metagenomics and reported that, of the 207 exogenous and intrinsic host factors investigated, 60% correlated significantly with gut microbiota (including 31 intrinsic factors, 12 diseases, 19 drug groups, four smoking categories and 60 dietary factors). Collectively, these factors explained 18.7% of the inter-individual variation in microbial composition [4]. These studies demonstrate that a wide range of health and environment factors associate with changes in the composition and functionality of the gut microbiota.

### A role for the gut microbiota in diabetes prevention and treatment

From an evolutionary perspective, the rapid increase in the prevalence of type 2 diabetes worldwide is related to rapid environmental changes that have had a negative impact on risk factors of diabetes, such as dietary habits and sedentary time. Although the composition of gut microbiota is affected by genetic and environmental factors, unless marked environmental changes occur, it is rather stable in adult (‘middle-aged’) individuals. Nonetheless, inter-individual variation in gut microbial composition is remarkably high. Given the plethora of biological functions that are regulated by the gut microbiota, and that its composition is dependent on adjustable factors (e.g. diet or drugs), new evidence for the potential use of the modifiable capacity of the gut microbiota in the development of better prevention and treatment options for type 2 diabetes are emerging.

**Fig. 1:**
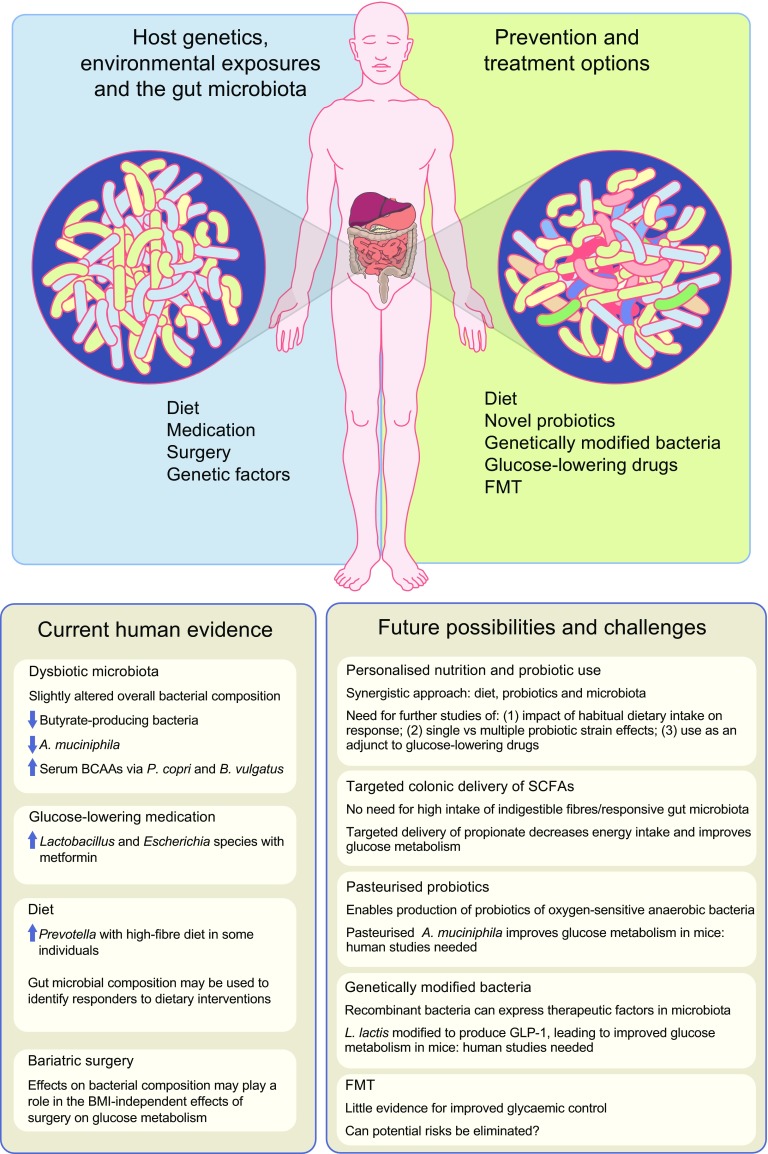
The gut microbiome as a prevention and treatment target for hyperglycaemia in type 2 diabetes: current evidence from human studies and future possibilities. Research has aimed to investigate how host genetics and internal and external environmental exposures (e.g. diet, medication, surgery) affect the gut microbiota, and how this information, along with information on gut bacterial composition, may be used to develop prevention and treatment strategies for personalised medicine in type 2 diabetes. Current human evidence has revealed that individuals with type 2 diabetes have a slightly altered overall bacterial composition, including decreased abundance of butyrate-producing bacteria and those with prediabetes/type 2 diabetes have a decreased abundance of *A*. *muciniphila*. Furthermore, in individuals with insulin resistance, serum BCAAs are shown to be elevated in insulin resistance and gut microbiota are thought to be a significant contributor, with *P*. *copri* and *B*. *vulgatus* being the main producers of BCAAs. Interestingly, glucose-lowering treatments, such as metformin, can alter overall bacterial composition e.g. by increasing abundance of *Lactobacillus* and *Escherichia* species. Diet has also been shown to cause short- and long-term alteration in gut microbial richness and composition. For example, high fibre intake increases *Prevotella* abundance. However, response to diet is varied, with some individuals being non-responders to dietary interventions. In line with this, gut microbial composition may provide a tool for the identification of individuals who are expected to benefit from dietary interventions. Further, evidence from bariatric surgery suggests that its effects on bacterial composition in the gut may contribute to the BMI-independent effects on glucose metabolism after surgery. Together, these findings open doors for novel future possibilities for the prevention and treatment of type 2 diabetes; however, the implementation of these is associated with many challenges. For example, a personalised nutrition approach (a synergistic approach involving diet, probiotics and microbiota) may be developed to improve glycaemic control. However, prior to implementing this strategy, there is a need for prospective follow-up studies, studies investigating the influence of habitual dietary intake on responsiveness to personalised nutrition, and large long-term randomised controlled trials (RCTs) to investigate single vs multiple probiotic strain effects and the use of probiotics as adjuncts to glucose-lowering drugs. Targeted colonic delivery of SCFAs may also be used to beneficially alter glycaemic control, without the need for high consumption of indigestible fibres (and for responsive gut microbiota, which may not be available in diabetes). In line with this, targeted delivery of propionate has been shown to decrease energy intake and improve glucose metabolism, making this a promising approach. Pasteurised probiotics provide another potential therapy in diabetes; these enable use of oxygen-sensitive anaerobic bacteria in diabetes treatment. For example, pasteurised *A*. *muciniphila* has been shown to improve glucose metabolism in mice. However, human studies are needed. Likewise, genetically modified bacteria can be designed to express therapeutic factors and incorporated into the microbiota. For example, *L*. *Lactis* (a ‘safe’ bacteria) has been genetically modified to produce GLP-1 and, hence, improve glucose metabolism in mice; however, human studies are lacking. Finally, FMT has been shown to be effective in the treatment of severe forms of *C*. *difficile* infection (for which there are high mortality rates if not treated). However, evidence is required to ensure that it can be used to improve glycaemic control in diabetes and that the potential risks of this therapeutic approach can be eliminated

In a review in 2007, Cani et al were the first to mention gut microbiota in the context of type 2 diabetes [5]. To date, a search of PubMed (www.ncbi.nlm.nih.gov/pubmed/, accessed 16 March 2017) using ‘type 2 diabetes (or hyperglycaemia) and gut microbiota (or gut microbiome)’ produces a list of over 600 papers that have been published in this area, of which almost 50% are reviews. Yet, despite there being some excellent (recent) reviews on this topic [6], it is challenging to identify the human evidence from the much larger number of animal studies in the field. Similarly, separating the findings related to obesity or inflammation from those related to glycaemic traits is not straightforward. Therefore, this review focuses on human studies and, when possible, on BMI-independent findings, aiming to converge the current and future possibilities and challenges of using the gut microbiota, and its plastic nature, in preventive and treatment strategies for type 2 diabetes (Fig. 1).

## Gut microbiota composition in type 2 diabetes and insulin resistance

The first study investigating the differences between human gut microbiota in individuals with type 2 diabetes and control participants was conducted by Larsen et al in 2010. Significant differences in the composition of microbiota at the phylum level were observed between the groups using a principal component analysis [7]. Thereafter, other studies have reported BMI-independent alterations in the gut microbiota of participants with type 2 diabetes, and although there has been a lack of consensus concerning which bacteria are significantly altered in type 2 diabetes, a common observation has been a decreased abundance of butyrate-producing bacteria with this condition [7–10]. More recently, Forslund et al challenged the question of whether type 2 diabetes-associated changes in the gut microbiota can be separated from those induced by commonly used blood glucose-lowering drugs [11]. They found that a large part of the previously observed differences in the gut microbiota in diabetes could indeed be explained by metformin treatment. In line with previous studies [10, 12], Forslund and colleagues found that individuals with type 2 diabetes who were not treated with metformin had less bacteria from genera known to produce butyrate as compared with control participants without diabetes. Meanwhile, they also elucidated that the previously reported increase in *Lactobacillus* species among type 2 diabetes individuals results from metformin treatment [11]. Metformin-treated individuals were also observed to have increased abundance of *Escherichia* species in the gut, suggested to potentially contribute to the adverse gastrointestinal effects of metformin.

Another recent study integrated data on insulin resistance, the gut microbiome and the fasting serum metabolome of 277 Danish individuals without diabetes to investigate whether the gut microbiome impacts upon insulin resistance-associated metabolic signatures. The serum metabolome of individuals with insulin-resistance was characterised by an increased potential for branched-chain amino acid (BCAA) biosynthesis and reduced potential for BCAA transport into bacterial cells [13]. The finding that BCAAs tightly co-varied with fasting serum metabolites of microbial origin, and that BCAA synthesis was driven by a few specific bacterial species (*Prevotella copri* and *Bacteroides vulgatus*; ‘top producers’ of BCAA), suggested that the gut microbiota could be an important source of elevated BCAAs in insulin resistance [13, 14].

One specific bacteria that has been the focus of several obesity and type 2 diabetes studies is the mucus-colonising *Akkermansia muciniphila*. Zhang et al found *A*. *muciniphila* to be decreased in individuals with prediabetes (impaired glucose tolerance and/or impaired fasting glucose) and newly diagnosed type 2 diabetes, and suggested that low abundance of this bacteria could be a biomarker for glucose intolerance [8]. More recently, *A*. *muciniphila* was observed to be decreased prior to the onset of type 2 diabetes in twins [9], while high abundance was associated with a healthier metabolic status in overweight/obese humans, as well as with greater improvements in glucose homoeostasis and body composition after energy restriction [15].

## Current prevention and treatment strategies for hyperglycaemia in type 2 diabetes by gut microbiota modification

### Modification of gut microbiota by diet and prebiotics

A number of studies have demonstrated that the gut microbial richness and composition are, to a large extent, modulated by diet [16, 17]; there is increasing evidence that insufficient consumption of indigestible carbohydrates may have led to a loss of bacterial species in the human gut that are reliant on these substrates and, as a consequence, there is decreased production of their fermentation end-products, short-chain fatty acids (SCFA) [18]. Epidemiological studies have been consistent in showing inverse association between dietary fibre and the incidence of type 2 diabetes, and dietary fibre and whole grains have been shown to increase the diversity of the human gut microbiota [19, 20]. In several studies, high fibre intake has been shown to associate with increased levels of the bacterial genus *Prevotella* [16, 17, 21]. Further, Kovatcheva-Datchary et al observed that improvements in postprandial glucose and insulin response after a 3-day intervention with barley kernel bread were dependent on the enrichment of *P. copri* in the microbiota of participants, and this enrichment was functionally connected to increased efficiency to digest complex polysaccharides in the barley kernel bread. Interestingly, for those whose glucose metabolism was not improved following the intervention, their gut microbiota was found to be neither enriched with *P*. *copri* nor was its potential to ferment complex polysaccharides increased following the trial. These findings suggest that analysis of the gut microbiota could be used to gain an understanding of individual responses to dietary interventions [22].

### The therapeutic effects of metformin are mediated by gut microbiota

The primary site of action and the main glucose-lowering effects of metformin have been debated. However, important novel information has now emerged demonstrating that the primary effects of the drug may reside in the gut; the pharmacological action of metformin was observed to include alterations in bile acid recirculation and gut microbiota, resulting in enhanced glucagon-like polypeptide-1 (GLP-1) secretion [23]. Further, a meta-analysis of 199 individuals with type 2 diabetes and 544 control participants without diabetes, stratified by treatment regiments, found that metformin had a significant effect on gut microbiota composition, such that individuals with type 2 diabetes who were on metformin could be identified based on compositional changes in their gut microbiota [11]. Functionally, the gut microbiota of metformin-treated individuals had increased potential to produce butyrate and propionate, while the untreated participants had enrichment of microbial genes involved in the degradation of glycine and tryptophan, and depletion of genes involved in threonine and arginine degradation. This is particularly interesting since glycine has previously been associated with insulin sensitivity [24], and glycine supplementation has been suggested as a treatment for glutathione deficiency and has been shown to improve insulin sensitivity in type 2 diabetes [25]. Further, increased abundance of *Escherichia* in metformin-treated individuals has been functionally attributed to the enrichment of virulence factors and genes associated with gas metabolism, indicating involvement of this genus in the intestinal adverse effects of metformin. The work by Forslund et al thus suggests potential microbiota-mediated mechanisms behind both the therapeutic and adverse effects of metformin [11].

Additional evidence and mechanistic insights supporting a predominant action of metformin in the lower gut have recently been provided by clinical studies testing the response to delayed-release metformin (MetDR) in healthy volunteers and participants with type 2 diabetes [26, 27]. The delayed release of this drug relies on a pH-dependent dissolution of the enteric coat of the tablet; this first occurs at pH 6.5 in the distal intestine, where the density of L cells is high, to maximise the gut-based mechanisms of the drug. Pharmacokinetic and dose-ranging studies have demonstrated that MetDR has a glucose-lowering efficacy comparable with that of extended- release metformin but with a 40% increase in potency, thus allowing for lower doses of MetDR to be used [27]. In line with this, two randomised trials demonstrated that MetDR resulted in glucose-lowering effects comparable with immediate-release metformin, despite significantly lower systemic exposure [26]. Large-scale Phase III clinical trials are now needed, as well as studies in individuals with renal impairment, to fully elucidate the efficacy and safety of MetDR. In addition, whether MetDR affects the gut microbiota in a similar manner to immediate-release metformin is currently unknown and must be investigated.

### Bariatric surgery remodels the gut microbiota composition

Bariatric surgery leads to fast and major weight loss, and many individuals with type 2 diabetes achieve normal glucose and insulin regulation within days after surgery, suggesting a BMI-independent effect [28]. Bariatric surgery leads to many important physiological alterations (for a recent review see [29]) including both short- and long-term changes in gut microbiota composition [29, 30]. *Proteobacteria* have been found to be increased in the microbiota of operated individuals and elevations in plasma bile acids and alterations in bile acid composition have been linked to the compositional changes of microbiota [30]. Both the changes in *Proteobacteria* and bile acids following bariatric surgery have been suggested to contribute to the BMI-independent surgical effects on glucose metabolism [28]. However, the precise molecular mechanisms underlying the many physiological effects of bariatric surgery still need to be determined and whether such information can lead to development of novel non-surgical interventions for the treatment of type 2 diabetes remains to be seen.

## Towards novel prevention and treatment strategies for type 2 diabetes: possibilities and challenges offered or inspired by gut microbiota studies

### Personalised nutrition, probiotics and targeted delivery of propionate

Inter-individual differences in the composition of gut microbiota have been shown to result in individualised responses to dietary fibre interventions [22, 31] and analysis of gut microbial composition has been proposed as a tool for the identification of individuals who are expected to benefit from dietary interventions [32–34]. A promising future approach could use a synergistic approach involving both diet and microbiota in the prevention and treatment of type 2 diabetes. However, prospective follow-up studies that use gut microbiota composition as a strategy to identify individuals who would benefit from a certain dietary intervention are obligatory to provide evidence for the effectiveness of this approach. In addition, studies investigating the influence of habitual dietary fibre intake on an individual’s responsiveness (and the responsiveness of their gut microbiota) to probiotic intervention are needed [35]. A further challenge will be to perform large, long-term randomised controlled trials to investigate the effect of single vs multiple probiotic strains on glycaemic control and to determine their usefulness as an adjunct to glucose-lowering drugs, whilst elucidating how such effects may be mediated by changes in gut microbiota ecology.

The increasing evidence for a beneficial role of SCFAs in appetite control and energy homeostasis has inspired the development of methods to increase colonic SCFA levels without the need for high consumption of indigestible fibres and, hence, a responsive gut microbiota for their effective fermentation. A novel targeted delivery system was recently developed based on an inulin-propionate ester capable of releasing gram quantities of propionate in the proximal colon [36]. Such targeted delivery of propionate was demonstrated to acutely reduce energy intake and ameliorate long-term weight gain in overweight adults [37]. In addition, 24 weeks of supplementation with the inulin-propionate ester significantly improved acute insulin secretion and beta cell function, suggesting that increasing colonic propionate improves glucose metabolism, both via acute elevations in GLP-1 and peptide YY (PYY) [36] and by long-term stimulatory actions of propionate on beta cells [37].

### Pasteurisation and identification of the therapeutic components of probiotics

The use of *A*. *muciniphila* in humans is hampered by its high sensitivity to oxygen and its requirement for mucus-based medium that is incompatible with human administration. To circumvent these hurdles, in a recent study, Plovier et al grew this bacterium on a synthetic medium where mucus was replaced with compounds appropriate for human administration. Further, Plovier and colleagues were able to produce a non-replicative, pasteurised preparation of *A*. *muciniphila* [38], which was surprisingly found to have an enhanced capacity to improve glucose metabolism in mouse models of obesity and diabetes. Further, a specific outer membrane protein of *A*. *muciniphila*, Amuc_1100, was found to partly recapitulate the bacteria’s beneficial effects. Preliminary human data indicated that, following 2 weeks of treatment, when grown on the synthetic medium both the pasteurised and the live bacterium were safe for human administration [38]. However, the efficacy of *A*. *muciniphila* preparations as a potential therapeutic tool for diabetes remains to be shown.

### Faecal microbiota transplantation

Faecal microbiota transplantation (FMT) is a technique where case or control microbiota is transplanted into germ-free mice to study the effect of faecal microbiota independent of other environmental and microbial interactions. FMT is commonly used to prove causality, however, whether causality can be effectively determined via this method is debated [39]. For example, only a subset of human gut microorganisms is capable of colonising the gut of germ-free mice [39, 40] and, in particular, many potential butyrate-producing genera may be poorly transferred [39]. Nonetheless, the fact that individuals with severe *Clostridium difficile* infection are successfully treated with FMT has led researchers to hypothesise that transplantation of healthy faecal microbiota could cure metabolic disease. Testing this hypothesis in mice has provided promising results [41]. In the first human study of 18 individuals, FMT from lean individuals to men with the metabolic syndrome resulted in significant improvements in peripheral insulin sensitivity along with an increase in butyrate-producing bacteria in the recipient’s faecal microbiota [42], which is in line with observational studies where butyrate-producing bacteria have been observed to be enriched in the faeces of healthy individuals [12]. However, we must emphasise that this study was small, it did not report data on glucose levels during the intervention and it has not yet been replicated. Moreover, not all participants responded to FMT, again raising the question as to why some individuals are responsive while others are not [22]. Thus, the current evidence for FMT as a therapeutic tool to improve glycaemic control and/or insulin sensitivity is very limited. In addition, this method is still very new and, hence, much more research must be devoted to FMT to explore the potential risks related to its impact on the vast physiological functions regulated by gut microbiota and to eliminate the threat of transplantation of pathogenic bacteria.

### Genetically modified microbiota

One novel strategy to alter the microbiome is to incorporate genetically modified bacteria that express therapeutic factors into microbiota. Recently, a recombinant *Lactococcus lactis* strain that was genetically modified to produce GLP-1 was shown to stimulate insulin secretion and improve glucose tolerance in mice [6]. *L*. *lactis* is used in many fermented food products and is generally regarded as safe and, despite the lack of human evidence for its ability to treat diabetes by acting as a host for the production, secretion and delivery of incretins (or other compounds) directly to the intestine, recombinant bacteria that produce IL-10 have been shown to be safe in a Phase I clinical trial in participants with Crohn’s disease [43]. Other studies in mice have demonstrated that therapeutic compounds produced by genetically modified colonic bacteria can have additional therapeutic effects in tissues beyond the intestinal tract, such as the liver [44]. Compared with wild-type probiotics, the use of genetically modified gut microbiota has clear advantages as it enables selection of an effective ‘coloniser’ as the carrier bacteria, and production of a desired therapeutic compound. Further, the recombinant bacteria could be designed to respond to specific temporal signals, like dietary factors [44].

### Multi-omics in gut microbiota research

During the last decade genome-wide association studies (GWAS) have revolutionised our understanding of genetic factors affecting multifactorial diseases in humans, including identification of over 120 loci that associate with type 2 diabetes or glycaemic traits. The gut microbiome essentially complement our coding potential and have also been named our ‘second genome’ [45]. Indeed, based on metagenomic sequencing data of faecal samples from 1267 individuals, a catalogue of human faecal microbiome was created including 9,879,896 genes, which is almost 500 times the number of human genes (~20,000). However, each sample contained an average of 762,665 bacterial genes, thus representing around 40 times more genes as compared with the human genome [45]. In 2014, Goodrich et al assessed the heritability of the human gut microbiota in a twin study and demonstrated that human genetic variation influences the microbial phenotype [46]. More recently, the same research group tripled the sample size to 1126 twin pairs and compared the differences in heritability of 945 widely shared bacterial taxa in monozygotic vs dizygotic twin pairs. Their findings indicated that 8.8% of all taxa have heritability estimates of greater than 0.2, meaning that at least 20% of the variance in the abundance of these 8.8% of taxa could be attributed to genetic differences. *Christensenellaceae* was identified as the most heritable taxa [47]. The first human GWAS of gut microbiota composition observed a significant association between variation in the gene *PLD1*, which has been associated with obesity in African-American individuals, and the abundancy of *Akkermansia* [48]. Another GWAS found nine SNPs to be associated with specific taxa, and replicated an earlier finding that an interaction between the *LCT* gene and *Bifidobacteria* was dependent on dairy intake [49]. This study provided the first example of how dietary intake can modify the association between a genetic variant related to metabolic disease and the abundance of specific gut bacteria. For GWAS, the size of these studies is very small and, thus, much better powered studies are warranted. This is especially true for GWAS in gut microbiota research since, compared with GWAS in other areas, there are a vast number of variables (i.e. a huge array of possible microbial phenotypes), increasing the multitude of tests that are required [50].

Another future opportunity in gut microbiota research is microbial metagenomic GWAS, which, so far, has only been used in the study of pathogenic microorganisms [51]. Although a number of analytical advances are required to handle the unique features of microbial genomics, such an approach could provide exciting new insights into the effects of microbial variants and, if integrated with human genomic data, how they may be dependent on the human genetic background. Arguably, for microbiota-based personalised treatments to become a successful future reality, integration of human multi-omic data (such as genetics, transcriptomics, epigenetics, proteomics and metabolomics) with microbiome data (such as strain-level variation, transcriptomics, proteomics and metabolomics) in large, longitudinal cohorts will most likely be required [52].

## Conclusion

The WHO has predicted type 2 diabetes to be the seventh leading cause of death by 2030 and there is an urgent need for the development of more efficient prevention and treatment strategies. The metabolic potential of the gastrointestinal tract and its microbiota are increasingly recognised as promising targets to improve glycaemic control and treat type 2 diabetes. Importantly, recent data has demonstrated that the glucose-lowering effects of metformin are mediated by changes in the composition and function of gut microbiota. Several potential gut-targeting glucose-lowering treatment strategies are now emerging and show initial promise, yet a better understanding of the mechanisms underlying these effects in humans is required. So far, human studies in this field have been limited in size and the results have often been inconclusive and difficult to interpret. Therefore, to fully elucidate the modifiable capacity of the gut microbiota and its potential for use in the prevention and treatment of type 2 diabetes, large longitudinal and interventional studies are required. Another great challenge for the future is the personalisation of nutrition to allow for individual dietary advice in diabetes, taking into account gut microbiota, drug use, genetics and relevant environmental factors (here probiotics might provide an opportunity to enhance the beneficial health effects of diet or glucose-lowering drugs). However, methodologies must be developed to enable integration of multi-omics data of both human and microbial origin for this to happen. In addition, many other methodological challenges remain in this area of research, from appropriate standardisation of the DNA-extraction methods used, to functional characterisation of bacterial genes with unknown functions and improvement of the statistical and bioinformatic processing of this complicated data. Hence, advancing our knowledge on the role of gut microbiota in type 2 diabetes and using this knowledge to enable a personalised medicine approach for the treatment and management of type 2 diabetes will presumably require long, painstaking work with no scope for short cuts.

## Electronic supplementary material

ESM 1 (PPTX 225kb)

ESM Table 1 (PDF 106 kb)

## Abbreviations

BCAA: Branched-chain amino acid
FMT: Faecal microbiota transplantation
GLP-1: Glucagon-like polypeptide-1
GWAS: Genome-wide association study
MetDR: Delayed-release metformin
SCFA: Short-chain fatty acid
