# Supplementary material for: The gut microbiome as a target for prevention and treatment of hyperglycaemia in type 2 diabetes: from current human evidence to future possibilities
Source: Diabetologia. 2017 Apr 22;60(6):943–51. doi: 10.1007/s00125-017-4278-3 (PMC5423958; doi:10.1007/s00125-017-4278-3)
Supplement: Supplementary file 1 — (PPTX 225kb) [file 125_2017_4278_MOESM1_ESM.pptx]

## Slide 1
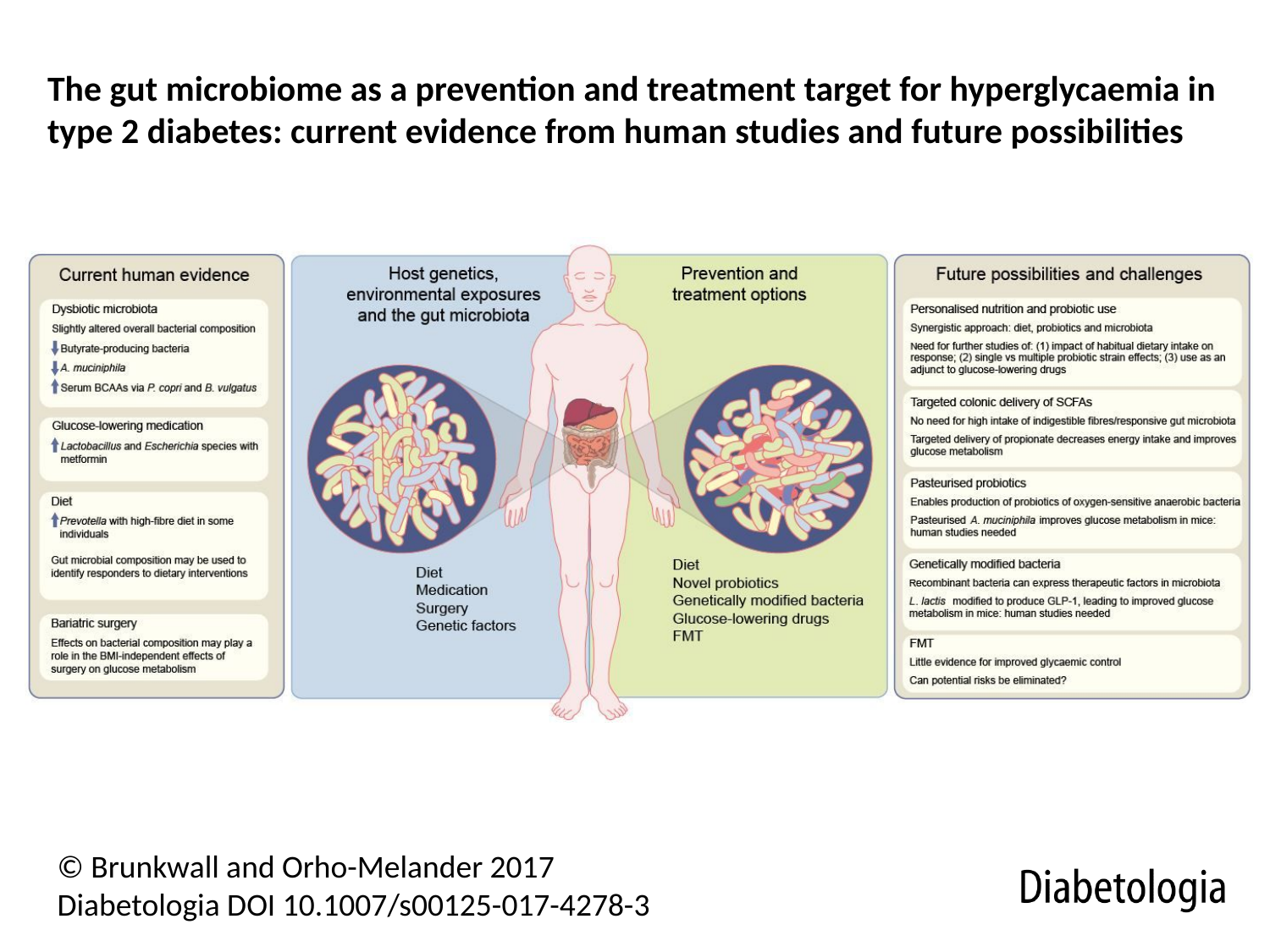

The gut microbiome as a prevention and treatment target for hyperglycaemia in type 2 diabetes: current evidence from human studies and future possibilities
© Brunkwall and Orho-Melander 2017
Diabetologia DOI 10.1007/s00125-017-4278-3
