# Supplementary material for: The gut microbiome as a target for prevention and treatment of hyperglycaemia in type 2 diabetes: from current human evidence to future possibilities
Source: Diabetologia. 2017 Apr 22;60(6):943–51. doi: 10.1007/s00125-017-4278-3 (PMC5423958; doi:10.1007/s00125-017-4278-3)
Supplement: Supplementary file 2 — (PDF 106 kb) [file 125_2017_4278_MOESM2_ESM.pdf]

Gut microbiome as a prevention and treatment target for hyperglycaemia of type 2 diabetes: from current human evidence to future possibilities

ESM Table1 – Sequencing methods used in the studies referred

Reference number	First author	Title	Sequencing method
3	Faloney	Population-level analysis of gut microbiome variation	16S sequencing
4	Zherenokova	Population-based metagenomics analysis reveals markers for gut microbiome composition and diversity	Metagenomic sequencing
7	Larsen	Gut microbiota in human adults with type 2 diabetes differs from non-diabetic adults	16S sequencing
8	Zhang	Human gut microbiota changes reveal the progression of glucose intolerance	16S DNA
10	Karlsson	Gut metagenome in European women with normal, impaired and diabetic glucose control	Metagenomic sequencing
11	Forslund	Disentangling type 2 diabetes and metformin treatment signatures in the human gut microbiota	Metagenomic sequencing
12	Qin	A metagenome-wide association study of gut microbiota in type 2 diabetes	Metagenomic sequencing
13	Pedersen	Human gut microbes impact host serum metabolome and insulin sensitivity	Metagenomic sequencing
15	Dao	Akkermansia muciniphila and improved metabolic health during a dietary intervention in obesity: relationship with gut microbiome richness and ecology	Metagenomic sequencing
16	David	Diet rapidly and reproducibly alters the human gut microbiome	16S sequencing
19	Tap	Gut microbiota richness promotes its stability upon increased dietary fibre intake in healthy adults	16S sequencing
20	Martinez	Gut microbiome composition is linked to whole grain-induced immunological improvements	16S sequencing
21	De Filippo	Impact of diet in shaping gut microbiota revealed by a comparative study in children from Europe and rural Africa	16S sequencing
22	Kovatcheva-Datchary	Dietary fiber-induced improvement in glucose metabolism is associated with increased abundance of Prevotella	16S sequencing
20	Tremaroli	Improved glucose metabolism following bariatric surgery is associated with increased circulating bile acid concentrations and remodeling of the gut microbiome	Metagenomics sequencing
32	Korpela	Gut microbiota signatures predict host and microbiota responses to dietary interventions in obese individuals	16S sequencing
33	Le Chatelier	Richness of human gut microbiome correlates with metabolic markers	Metagenomics sequencing
34	Salonen	Impact of diet and individual variation on intestinal microbiota composition and fermentation products in obese men	16S sequencing and quantitative PCR
35	Healey	Influence of habitual dietary fibre intake on the responsiveness of the gut microbiota to a prebiotic: protocol for a randomised, double-blind, placebo-controlled, cross-over, single-centre study	16S sequencing
39	Zhang	Environmental spread of microbes impacts the development of metabolic phenotypes in mice transplanted with microbial communities from humans	16S sequencing

Gut microbiome as a prevention and treatment target for hyperglycaemia of type 2 diabetes: from current human evidence to future possibilities

42	Vrieze	Transfer of intestinal microbiota from lean donors increases insulin sensitivity in individuals with metabolic syndrome	HITChip
47	Goodrich	Genetic determinants of the gut microbiome in UK twins	16S sequencing
48	Davenport	Genome-wide association studies of the human gut microbiota	16S sequencing
49	Bonder	The effect of host genetics on the gut microbiome	Metagenomic sequencing
